# Pixel-tracking derived strain using the GlasgowHeart Method

**DOI:** 10.1186/1532-429X-18-S1-P9

**Published:** 2016-01-27

**Authors:** Kenneth Mangion, Hao Gao, Aleksandra Radjenovic, Xiaoyu Luo, Caroline Haig, Colin Berry

**Affiliations:** 1 BHF Glasgow Cardiovascular Research Centre, University of Glasgow, Glasgow, UK; 2 School of Mathematics and Statistics, University of Glasgow, Glasgow, UK; 3 Robertson Centre for Biostatistics, University of Glasgow, Glasgow, UK

## Background

Estimation of strain parameters from cine acquisitions, such as balanced steady state free precession (b-SSFP) is advantageous, as it would obviate the need for acquisition of additional strain sequences reducing scanning time and making strain more accessible to clinicians. 2D strain derived from feature-tracking is now commercially available. The GlasgowHeart cine-strain method is designed to overcome some limitations of currently available feature-tracking methods by estimating pixel-wise strain for myocardial deformation incorporating all of the myocardial tissues. The aims of this pilot study was to ensure that 2D peak circumferential strain estimated from the GlasgowHeart method is feasible in healthy volunteers (n = 20) and reproducible with minimal intra- and inter- observer variability.

## Methods

Healthy volunteers aged at least 18 years of age with no prior medical history were invited to participate. A subset of 20 healthy adult volunteers underwent 1.5T CMR twice, < 2 days apart. Written consent was obtained. Mid-LV cine sequences, were analysed with the GlasgowHeart software. The process involves contouring the myocardial borders at end-diastole and segmenting the myocardium by using the right ventricular insertion point according to the 16 segment AHA model. Two observers independently analysed 40 short axis slices using the cine-strain method for inter-observer variability. One observer re-analysed the 40 short axis slices 10 days later for intra-observer variability. Scans were analysed in a random order. Pearson correlation and Bland-Altman analysis were used to analyse the data.

## Results

20 participants were used in the subset analysis (mean age ± SD 49.5 years (17.2) 50% male). Peak circumferential strain (Ecc) measured on the first set of MRIs by the two observers (Figure 2A,B) was highly correlated (R = 0.915, p < 0.001) and in excellent agreement (mean difference = 0.01; 95% LoA: -0.01, 0.02). The repeated image analysis (Figure 2C,D) also disclosed a high degree of association in paired measurements of Ecc that was strongly correlated(R= 0.915, p< 0.001) and in excellent agreement (mean difference = 0.00; 95% LoA: -0.02, 0.01). Ecc measured in the second set of MRIs by 2 observers was well correlated (R = 0.937, p < 0.001) and in excellent agreement (mean difference = 0.00; 95% limits of agreement were -0.016 and 0.021). The repeated image analysis at follow-up yielded Ecc that was well correlated(R= 0.942, p < 0.001) and in excellent agreement (mean = 0.00; 95% LoA: -0.009 and 0.009). There was no difference between the average global Ecc at different time points (p > 0.05).

**Figure 1:**
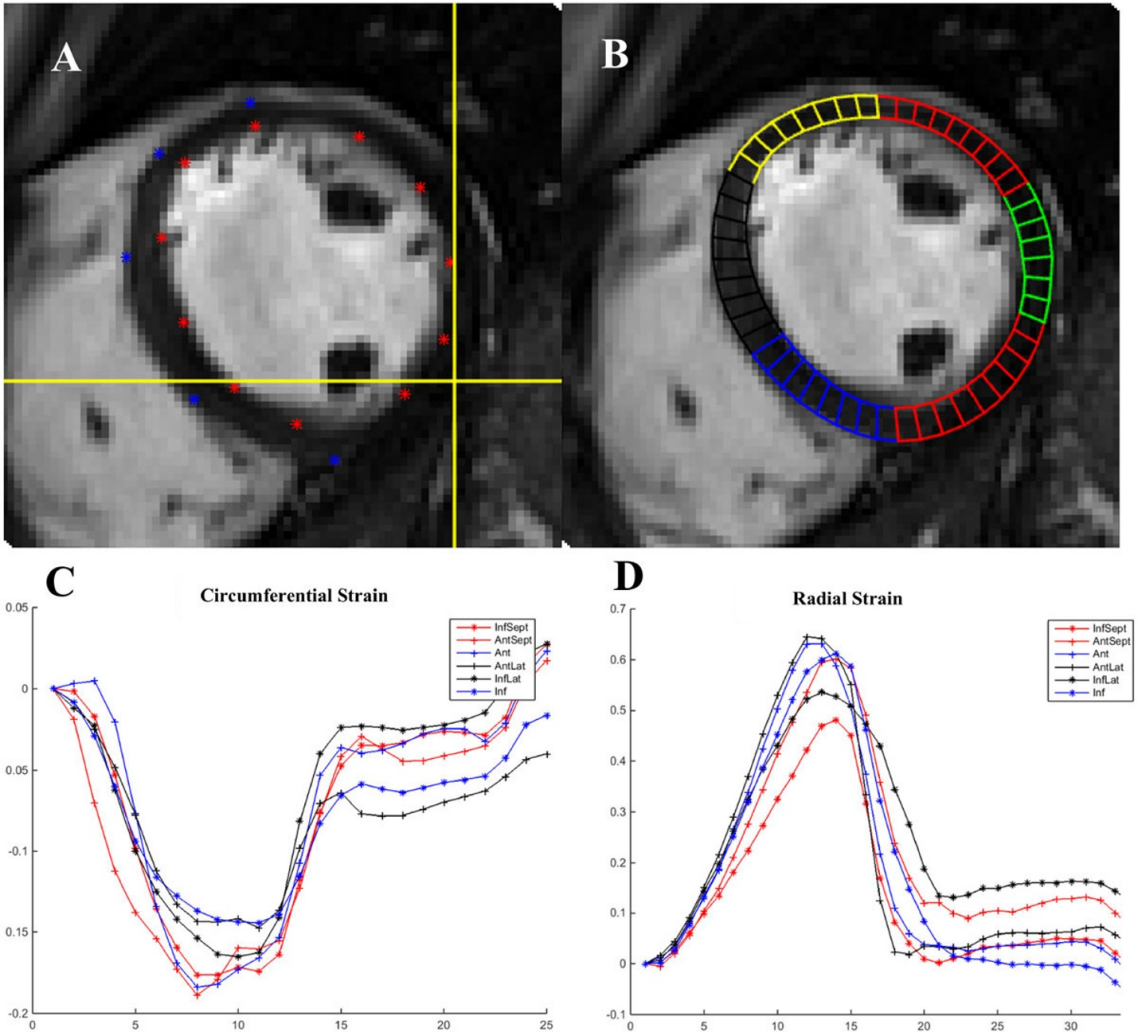
**The GlasgowHeart pixel-tracking software**. (A) shows border delineation (B) shows automatic segmentation using the inferior right ventricular insertion point. (C) and (D) show graphical output as circumferential and radial strain graphs, respectively.

**Figure 2:**
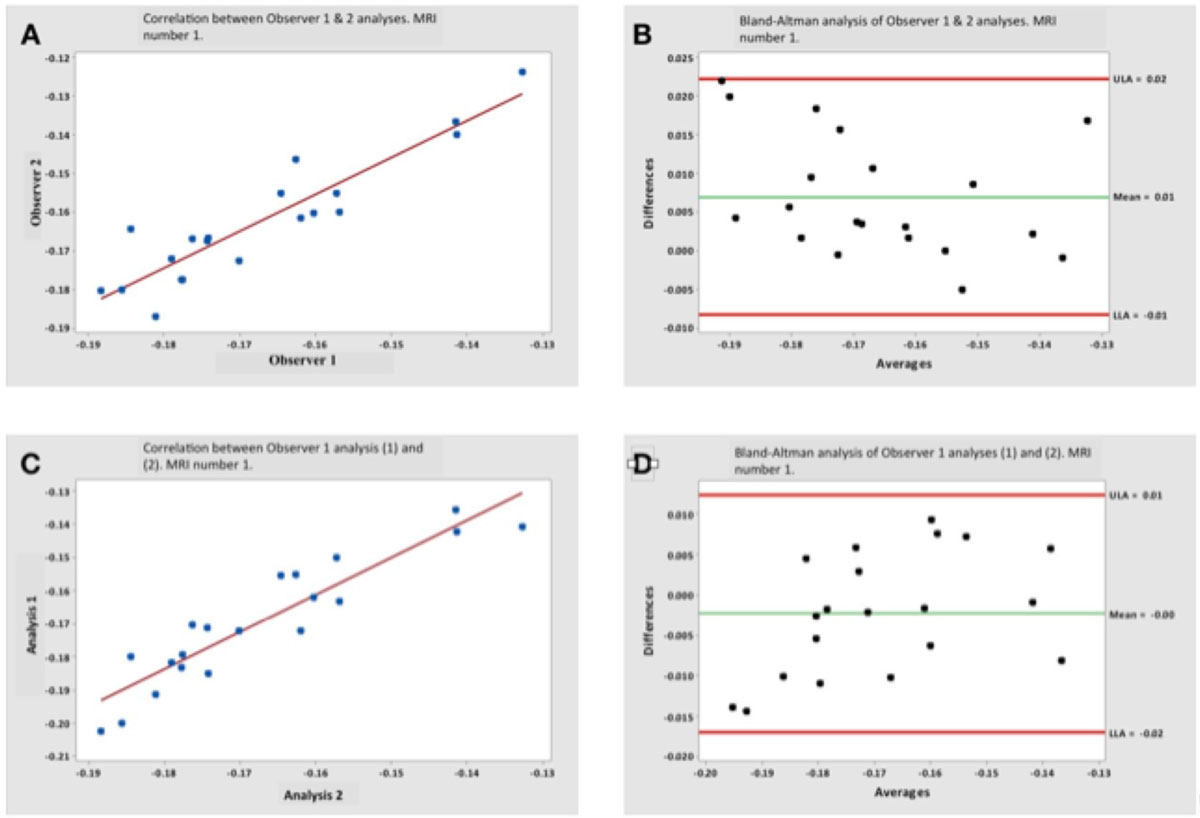
**Inter- and intra- observer variability, first CMR set**. (A) correlation analysis of global Ecc strain analysed by Observers 1 and 2 (B) Bland-Altman analysis of the results for inter-observer variability (C) correlation analysis of the 2 sets of analysis by Observer 1 (D) Bland-Altman analysis of the results for intra-observer variability.

## Conclusions

The results presented demonstrate that the GlasgowHeart method is a robust and reproducible method of assessing cine-derived circumferential strain. By tracking a higher proportion of voxels than the currently available feature tracking software, it has a clear potential to provide a more accurate assessment of strain.

